# Decoding plasma cell maturation dynamics with BCMA

**DOI:** 10.3389/fimmu.2025.1539773

**Published:** 2025-03-11

**Authors:** Sebastian R. Schulz, Shannon R. Menzel, Jens Wittner, Carolin Ulbricht, Alina T. Grofe, Edith Roth, Ritu Mann-Nüttel, Stefanie Scheu, Andrew J. Kueh, Alexander Jäck, Marco J. Herold, Anja E. Hauser, Katharina Pracht, Wolfgang Schuh, Hans-Martin Jäck

**Affiliations:** ^1^ Division of Molecular Immunology, Internal Medicine 3, University Hospital Erlangen, Nikolaus-Fiebiger Center, Friedrich-Alexander-University Erlangen-Nürnberg, Erlangen, Germany; ^2^ Charité-Universitätsmedizin Berlin, Corporate Member of Freie Universität Berlin and Humboldt-Universität zu Berlin, Department of Rheumatology and Clinical Immunology, Berlin, Germany; ^3^ Deutsches Rheuma-Forschungszentrum (DRFZ) Berlin, a Leibniz Institute, Berlin, Germany; ^4^ Institute of Chemistry and Biochemistry, Department of Biology, Chemistry and Pharmacy, Freie Universität Berlin, Berlin, Germany; ^5^ Institute of Medical Microbiology and Hospital Hygiene, Medical Faculty and University Hospital Düsseldorf, Heinrich Heine University of Düsseldorf, Düsseldorf, Germany; ^6^ Institute of Immunology, University Medical Center Rostock, Rostock, Germany; ^7^ Olivia Newton-John Cancer Research Institute, Melbourne, VIC, Australia; ^8^ School of Cancer Medicine, La Trobe University, Melbourne, VIC, Australia; ^9^ The Walter and Eliza Hall Institute of Medical Research, Melbourne, VIC, Australia; ^10^ Department of Medical Biology, University of Melbourne, Melbourne, VIC, Australia; ^11^ Department of Neurology University Hospital, LMU Munich, Munich, Germany

**Keywords:** plasma cells, thymus, bone marrow, survival, antibody-secreting cells (ASC), BCMA (TNFRSF17), spleen

## Abstract

Plasma cells provide protective antibodies following an infection or vaccination. A network of intrinsic and extrinsic factors fine-tunes the generation of a heterogenous plasma cell pool with varying metabolic requirements, transcriptional profiles and lifespans. Among these, the B cell maturation antigen (BCMA) has been implicated in the APRIL-mediated survival of long-lived plasma cells. To characterize the terminal maturation of plasma cells, we constructed a BCMA reporter mouse (BCMA:Tom) that exclusively labeled antibody-secreting cells and revealed that BCMA:Tom expression varied by IgH isotype and increased with plasma cell maturity. The BCMA reporter, used alongside the Blimp1-GFP reporter, also allowed detailed tracking of plasma cell development and highlighted the importance of the *in vivo* microenvironment to complete plasma cell maturation. Therefore, the BCMA:Tom reporter mouse provides a valuable tool for tracking plasma cell development and maturation with flow cytometry or advanced imaging techniques, enabling a deeper understanding of the mechanisms regulating plasma cell heterogeneity and longevity.

## Introduction

Plasma cells, the terminally differentiated effectors of the B cell lineage, are vital for the humoral immune response and memory against pathogens. These cells can persist in specialized niches in bone marrow or intestines for months to years in mice and humans (1–3). However, the molecular mechanisms driving their generation and long-term survival are not fully understood. Plasma cells differentiate from activated B cells via proliferating antibody-secreting plasmablasts into resting plasma cells with varying lifespans. This differentiation process involves a series of morphologic, phenotypic and functional changes controlled by external stimuli provided by the tissue environment and an intrinsic network of transcription factors, with a key role for *Irf4* and Blimp1 (*Prdm1)* in establishing plasma cell identity (4, 5). In addition, the fate decision of antigen-activated B cells to develop into short- or long-lived plasma cells depends on the nature of the antigen and the microenvironment in which they are formed (6).

Among the described factors contributing to plasma cell persistence, the B cell maturation antigen (BCMA) encoded by the tumor necrosis factor receptor superfamily member 17 gene (*Tnfrsf17*) takes center stage as a proposed mediator of APRIL-dependent plasma cell survival (7) and a promising therapeutic target to treat plasma cell disorders including refractory multiple myeloma (8). While high *Tnfrsf17* expression has been detected in plasma cell transcriptomes, detailed analyses of BCMA abundance and its function in plasma cell biology have been hampered by the near complete cleavage of BCMA from the surfaces of murine plasma cells by γ-secretase (9).

To conduct an in-depth study of BCMA expression during plasma cell maturation, we developed and characterized a novel BCMA reporter mouse model (BCMA:Tom). This model employs a fluorescent tdTomato reporter gene under the control of the endogenous *Tnfrsf17* promoter. Combined with the established Blimp1-GFP reporter mouse (10), we found that BCMA:Tom is exclusively detected in antibody-secreting cells (ASCs), with its expression varying with Ig heavy chain (IgH) isotypes and plasma cell maturity, enabling the identification and characterization of newly formed and late plasma cell subpopulations. Therefore, the BCMA:Tom mouse significantly advances the toolkit for unraveling the complexities of differentiation and function of antibody-secreting plasmablasts and plasma cells.

## Materials and methods

### Mice

C57BL/6NRj mice were purchased from Janvier (Le Genest Saint Isle, France). Blimp1-GFP reporter mice were obtained from Dr. Nutt, WEHI, Melbourne, B cell-deficient JH^−/−^/CD8^−/−^ recipients from Thomas Winkler (Friedrich-Alexander University of Erlangen-Nürnberg), Ai9(RCL-tdT) mice with a ROSA26-flox-stop cassette from David Vöhringer (University Hospital Erlangen). All mice were maintained under pathogen-free conditions in the Franz-Penzoldt-Center animal facility of the University of Erlangen-Nürnberg. All animal experiments were performed according to institutional and national guidelines.

### Construction of transgenic mice

To generate BCMA:Tom reporter mice, the *Tnfrsf17* locus on mouse chromosome 16 was targeted using two single guide RNAs (sgRNAs) to stimulate homologous recombination (5’-CACGTGACAGATACCCTTAC-3’, 5’-GACACTGAGTGAGTCCGAGC-3’). A targeting vector containing homology arms of approximately 2 kilobases was constructed. This vector was designed to insert an IRES-tdTomato cassette between Exon 3 and the 3’ untranslated region (3’UTR) of the BCMA gene. Additionally, loxP sites, flanking Exon 3 and the 3’UTR of BCMA, were introduced to facilitate future conditional knockout strategies.

For establishing the BCMA:Cre line, a single sgRNA targeting the BCMA locus was used to create double-strand breaks to stimulate homologous recombination (5’-GCCGTAGTCACCCGTTTTTG-3’). The targeting vector, containing homology arms of approximately 2 kilobases, was utilized to introduce an IRES-Cre cassette downstream of the BCMA Exon 3 stop codon.

The sgRNAs, Cas9 protein and targeting vectors were injected into the pronucleus of fertilized one-cell stage embryos isolated from C57BL/6J breeders. These embryos were then transferred into pseudo-pregnant recipient mice. Viable pups born from the recipient mice were screened for integration of the constructs via PCR. Targeted animals were backcrossed twice to wildtype C57BL/6J mice to eliminate off-target mutations. The correct positional integration of the targeting vector was validated using long-range PCR. Primers were used for genotyping the BCMA wildtype (5’-GATCGGCTCAGCTGGACAAG-3’, 5’-CTTCACACCAGTTAGGAAGC-3’), BCMA:Tom (5’-GGACGAGCTGTACAAGTGATG-3’, 5’-TTGGTTGCCCTGGAACTAGC-3’) and BCMA:Cre (5’-ACGACCAAGTGACAGCAATG-3’, 5’-GCTAACCAGCGTTTTCGTTC-3’) loci. Heterozygous BCMA:Tom^wt/+^ and BCMA:Cre^wt/+^ were used in all experiments.

### B cell stimulations

Splenic B cells were isolated by negative selection with the EasySep Mouse B Cell Isolation Kit (StemCell, Cat.: 19854), according to the manufacturer’s instructions. eFluor450 labeling (eBiosicence; 65-0842-85) of purified B cells was performed as described (11). The isolated B cells were stimulated for three days with 10 µg/ml LPS (Sigma-Aldrich, Cat.: L3012) or with 10 µg/ml anti-CD40 (clone FGK4.5, BioXCell) and 100 U/ml mouse IL-4 (BioLegend, Cat.: 574304) and seeded in R10 medium (RPMI-1640 supplemented with 1 mM sodium pyruvate, 2 mM L-glutamine, 100 U/ml penicillin-streptomycin, 50 μM β-mercapto-ethanol, 10% fetal calf serum) at densities of 0.25x10^6^ cells/ml and 0.5x10^6^ cells/ml, respectively.

### iGB cell culture

40LB feeder cells (12) were kindly provided by Daisuke Kitamura (Tokyo University of Science, Japan). Feeder cells were irradiated with 100 Gy at 10^6^ cells/10 ml D10 medium. 5x10^5^ cells were seeded in 2 ml R10 medium + 10 mM HEPES in a 6-well plate one day before starting or re-seeding the iGB culture. Typically, 10^5^ isolated B cells were seeded onto the feeder cell layer in 4 ml R10 + 10 mM HEPES. IL-4 (0.1 U/ml, Milteny, Cat: 130-097-761) was added to the primary culture for 4 days. On day 4, the cells were re-plated onto a new feeder layer and cultured with rIL21 (10 ng/ml, Peprotech, Cat: 210-21). 3 days later, B cells were either re-plated on irradiated feeder cells (iGB21 culture) or cultured without feeder cells (iGB APRIL culture) in the presence of 50 ng/ml multimeric APRIL (AdipoGen, Cat: AG-40B-0089-C010) and 10 ng/ml mouse IL-6 (Peprotech, Cat: 216-16). B cells were harvested by carefully removing the supernatant and adding 2 ml PBS + 2%FCS + 2 mM EDTA for 5 minutes at RT. Live B cell numbers were quantified with the Nucleocounter3000 (Chemometec) after staining the cells with DAPI and acridine orange. Cells were cultured in a humidified atmosphere at 37°C with 5% CO_2_.

### Adoptive plasmablast transfer

Splenic B cells from Blimp1-GFP/BCMA:Tom reporter mice were isolated and stimulated with 10 µg/ml LPS with a cell density of 0.5x10^6^ cells/ml. Blimp1-GFP^+^ BCMA:Tom^−^ CD3^−^ cells were isolated on day 3 of stimulation on a Beckman Coulter MoFlo Astrios EQ. After washing the sorted cells with PBS, 5x10^6^ cells were transferred into JH^−/−^ CD8^−/−^ (13) recipients via retroorbital injection in a volume of 50 µl PBS. Recipient mice were sacrificed, and bone marrow single-cell suspensions were analyzed by flow cytometry.

### Production of recombinant anti-BCMA antibody and isotype control antibody

The sequence of an antibody (clone 25C2) binding both human and murine BCMA in ELISA was identified from patent literature (14). The VH and Vκ sequences were synthesized (IDT), cloned into expression vectors for human IgG1 and human Ckappa (15) and purified from 293F supernatants as described. TRES480 (16) was recombinantly produced according to the same protocol and used as the isotype control antibody. The specific binding of 25C2 to mouse BCMA was verified in 293T cells transiently transfected with an expression plasmid encoding a mouse BCMA-GFP fusion protein [Origene, Cat.: MG201716] (data not shown).

### ELISA

Blood was taken from the vena facialis and was transferred into BD microtainer^©^ blood collection tubes, incubated for 30 min at RT and centrifuged for 90 sec. at 13,000 x g at RT. Sera were diluted as follows: total IgG 1:10,000, total IgM/IgA 1:4000. For detecting serum Ig by ELISA, 96-well flat bottom plates were coated with 50 μl/well of a 1 μg/mL solution with goat anti-mouse IgM, IgG or IgA (SouthernBiotech, 1021-01, 1030-01, 1040-01) in ELISA coating buffer (15 mM Na_2_CO_3_ and 35 mM NaHCO_3_ in dH_2_O). Unspecific binding was blocked with PBS-2% FCS for 1h at RT. Sera dilutions in PBS-2% FCS were incubated at 4°C overnight or at RT for 2h. As detection antibodies, 50 µL/well HRP-coupled goat-*anti*-mouse IgM (0.3 µg/mL), IgG (1 µg/mL) or IgA (0.2 µg/mL) (Southern Biotech., clone 1021-01, 1030-01, 1040-01) were incubated for 1h at RT. The TMB Substrate Reagent Set (BC OptEIA™, Cat# 555214) was used following the manufacturer’s protocol. ELISA plates were measured and analyzed using the BioLegend Mini ELISA plate reader at 450 nm. Analysis was performed using the *“Four Parameter Logistic Curve” online data analysis tool, MyAssays Ltd., accessed in the time of 2021-2024*, http://www.myassays.com/four-parameter-logistic-curve.assay.

### Flow cytometry

Single-cell suspensions for flow cytometric analyses were prepared and stained as described (11). Briefly, bone marrow was flushed out of the femora and tibiae with PBS + 2% FCS using a syringe with a 25G needle. Spleen and mLN were minced and homogenized using a syringe plunger and a 70 µm cell strainer. Cell suspensions were depleted from RBCs using RBC Lysis buffer (BioLegend, Cat.: 420301) and incubated with Fc block (anti-mouse CD16/32, eBioscience, clone 93) for 5 min at room temperature (RT). Then, cell suspensions were stained with various combinations of the following antibodies: CD138-PE.Cy7 (BioLegend, clone 281-2), TACI-APC (eBioscience, clone eBio8F10-3), TACI-BV421 (BD, clone 8F10), CD3-APC (eBioscience, clone 145-2c11), B220-PerCPCy5.5 (eBioscience, clone Ra3-6b2), CD19-BV421 (BioLegend, clone 6D5), IgA-AF647 (Southern Biotech, 1040-31), IgM-FITC (Southern Biotech, 1020-02), human IgG-AF647 (Southern Biotech, 2048-31), CD38- PerCPCy5.5 (BioLegend, clone 90), GL7-FITC (BD, clone GL7), CD95-PE.Cy7 (BD, clone Jo2). For all flow cytometric analyses, live/dead cell discrimination was based on FSC-A/SSC-A characteristics and doublets were excluded based on FSC-A, FSC-W characteristics, 40LB feeder cells in the iGB cell cultures were gated out based on FSC/SSC characteristics. Samples were analyzed with a Gallios flow cytometer (Beckman Coulter). The data were evaluated using the Kaluza Analysis software (v2.2).

### RNA sequencing and analysis

Blimp1-GFP/BCMA:Tom 29–35-week-old female mice were immunized and boosted intramuscularly into both hind legs after 70 days with 25 µl mRNA-1273 vaccine diluted in 25 µl sterile PBS. Seven days after boosting, BCMA:Tom^−^ and BCMA:Tom^+^ P3 plasma cells (CD138^hi^/Blimp1-GFP^+^/IgA^−^/IgM^−^) were sorted on a MoFlo Astrios EQ (Beckman Coulter) from the bone marrow of the immunized Blimp1-GFP/BCMA:Tom mice. Sequencing libraries were prepared from total RNA (RNeasy micro-Kit, Qiagen) using the Clontech SMART-Seq v4 kit. The libraries were sequenced on an Illumina HiSeq X instrument (2x150bp) by Admera Health LLC (New Jersey, USA). The reads were aligned to the mouse reference genome (GRCm38.p6) using STAR (17), and gene-wise counts were established with featureCounts (18). Differential expression analysis was conducted using the R package edgeR (19). Genes with low expression were excluded with the filterByExpr function with default filtering criteria (min.count = 10, min.total.count = 15), and immunoglobulin transcripts were removed from the analysis. Libraries were normalized with the “TMM” method before testing for differential expression between the BCMA:Tom^+^ and BCMA:Tom^−^ samples with the exactTest function. Genes with a fold change > 2 and a false discovery rate ≤ 0.05 were determined as significant.

Hallmark gene sets were obtained from MSigDB (20) and gene sets for plasmablast and plasma cell signatures were manually curated from the indicated publications. The enrichment of gene sets was assessed with the pre-ranked GSEA implementation in clusterProfiler (21). Clonotypes were extracted from RNA sequencing data with MiXCR (22) as described (23).

### Tissue clearing protocol

Tissue clearing was performed using the MarShie protocol (24) modified for clearing of the intestine. To perform perfusion, mice were deeply anesthetized by an intraperitoneal injection of Ketamine (250 μg/g body weight) and Xylazine (25 μg/g body weight). Mice were transcardially perfused with 100 ml cold 0.01 M phosphate-buffered saline (1x PBS) followed by 30ml of ice-cold SHIELD perfusion solution consisting of 50% v/v SHIELD Epoxy (LifeCanvas Technologies, Cat.: SH-Ex), 25% v/v SHIELD Buffer solution (LifeCanvas Technologies, Cat.: SH-Bf), 4% v/v paraformaldehyde (Electron Microscopy Sciences, Cat.: 15713) and 21% v/v distilled water. The intestine was harvested and postfixed in 15 ml SHIELD Perfusion for 2 days on a rotating device (MACSmix, Miltenyi Biotec, 20 rpm). Next, we incubated the samples for one day in SHIELD OFF solution at 4°C, followed by SHIELD ON solution at 37°C the next day. After washing in 1x PBS, we used the SmartClear II Pro device (Life Canvas STM-SC2A) to delipidate the intestine at 42° for 3 days (current = 1500 mA, limit = 90 V). We decolorized the samples with 25% w/v Quadrol (N,N,N′,N′-Tetrakis(2-Hydroxypropyl)ethylenediamine) (Sigma-Aldrich Cat.: 122262-1L) in 1x PBS at 37°C, rotating at 20 rpm for 2 days. For refractive index matching, we incubated the samples in Easy Index (LifeCanvas Technologies, Cat.: EI-Z1001), with a refractive index of 1.47 for 2 days.

### Sample mounting for light sheet fluorescence microscopy

We carefully glued the small intestine on a lancet (B|Braun 1 × 2000 Solofix) and immersed the samples in a 5 × 10 × 45mm fluorescence cuvette, optical glass (msscientific Chromatographie-Handel GmbH) mounted in Easy Index RI 1.47. The small cuvette containing the sample was glued onto the sample holder and subsequently immersed in the bigger imaging cuvette (LaVision Biotec – a Milteny company, Bielefeld, Germany) filled with Cargille Immersion Oil Type LDF, RI 1.515. To acquire microscopic image stacks, we used an Ultramicroscope II (LaVision Biotec – a Milteny company, Bielefeld, Germany) coupled to an Olympus MVX10 zoom body, providing a zoom ratio from 0.63x -6.3x. We used a 2x dipping objective (Olympus MVPLAPO2XC/0.5 NA) fitted with a 5.7 mm working distance dipping cap. The Ultramicroscope II was equipped with an Andor Neo sCMOS Camera with a pixel size of 6.5 × 6.5 μm² and a LaVision BioTec Laser Module. We used the following filter sets for image acquisition: ex 488 nm, em 520/50 nm for autofluorescence; ex 561 nm, em 620/60 nm for tdTomato signal. We used the 4x zoom body, resulting in a total magnification of 8.6 and a z-step of 5 μm. We adjusted the laser power depending on the intensity of the signal to avoid saturation. Exposure time was set at around 300 ms. We used the dynamic focus settings whenever deemed appropriate. The light sheet width was adjusted between 80 and 90% for a homogenous illumination of the field of view.

### Light sheet image processing and analysis

16-bit grayscale TIFF images were acquired separately for each channel using the ImSpector Pro software (LaVision Biotec). Tiff stacks were converted (Imaris File Converter, Bitplane AG) into Imaris files (.ims) and stitched with Imaris Stitcher. We used Imaris x64 software (version 9.9.1) and optically sliced the tissue using the ortho tool.

### Two-photon imaging

Two-photon deep tissue imaging experiments of freshly isolated organs *ex vivo* (spleen slices, bone marrow from femurs cut into halves, whole mesenteric lymph nodes and small intestinal lamina propria pieces) were performed using a multi-photon laser-scanning microscope (TriMScope II, LaVision BioTec, Bielefeld, Germany) without a cover glass, as previously described (25). Tissues were placed onto Petri dishes fixed using 2 drops of tissue glue (Epiglu), immersed in PBS and imaged using an upright system. Excitation of GFP was performed at 900 nm using the Titanium: Sapphire (Ti: Sa) laser and tdTomato was excited at 1100 nm via the optical parametric oscillator (OPO). Detection of the fluorescence signals was accomplished with photomultiplier tubes in the ranges 460 ± 30 (Ti: Sa SHG), 525 ± 25 (OPO SHG), 593 ± 20 (tdTomato) and 655 ± 20 nm (autofluorescence). In all experiments, we focused the laser beams with an 20x objective lens (Apochromat water-immersion, NA=1.0, WD=2 mm, Zeiss, Jena, Germany).

### Immunofluorescence

Femurs were extracted from mice and cleaned of surrounding tissue. Bones were fixed in freshly prepared 4% paraformaldehyde (EMS, Cat.: 15710) for 4 hours at RT while protected from light. Fixed bones were decalcified through two sequential 12-hour incubations at 4°C in 20% EDTA (Roth, Cat.: 8043.2), followed by dehydration via two sequential 12-hour incubations in 15% sucrose solution (J.T Baker, JTB-0348346-0071) at RT. De-calcified bones were embedded in SCEM embedding medium (Section-Lab) and frozen at -80°C. Cryo-sections were prepared with a thickness of 8 µm and mounted on SuperFrost plus slides (Thermo Scientific, Cat.: J1800AMNZ). Thawed sections were fixed in 2% PFA for 20 minutes, washed three times for 15min in PBS/0.05% Tween20 (Roth, Cat.:9127.1) and dried. After blocking by incubation with PBS/2% BSA for 1 h at RT, slides were incubated overnight at RT in a wet chamber with primary antibodies diluted in PBS/2%FCS/0.05% Tween 20 [rabbit-anti-RFP (Rockland, Cat.: 600-401-379), goat-anti-mouse Kappa-AF647 (Southern Biotech, Cat.: 1050-31)]. After washing three times for 15 min in PBS/0.05% Tween20, slides were incubated with secondary antibodies (donkey-anti-rabbit IgG-PE [BioLegend, Cat.: 406421)] for 2 hours at RT. Slides were again washed three times in PBS/0.05% Tween20 containing DAPI (Roth, Cat.: 6335.2) and covered with Fluoshield^®^ (Sigma, Cat.: F6182-20ML). Immunofluorescence images were acquired with a Axioplan2 fluorescence microscope (Zeiss).

### Statistics

Statistical analyses were performed using Prism v9 (GraphPad). Prior to testing, normal distribution of values was assessed with the Shapiro-Wilk test. Depending on the experimental design, one-way or two-way ANOVA was used to compare sample groups, with a repeated-measures design applied for dependent observations. Multiple comparisons were performed using a two-way ANOVA with Šídák’s multiple comparisons test or by testing for linear trend in one-way ANOVA. Bar charts are displayed using mean ± SD. A p-value of ≤0.05 (*), <0.01 (**), <0.001 (***) or <0.0001 (****) was considered significant.

## Results

### BCMA expression is restricted to antibody-secreting cells

**Figure 1:**
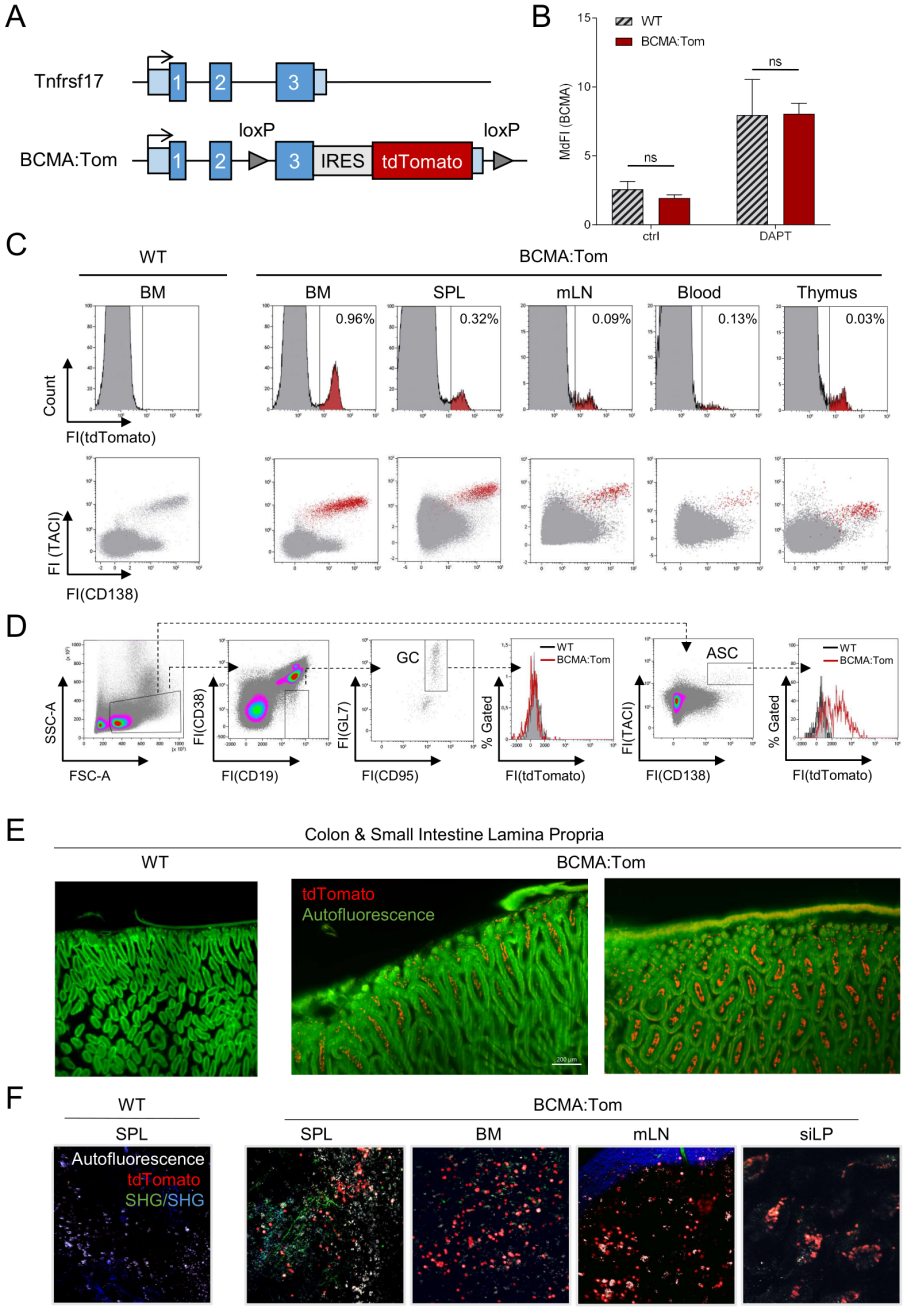
BCMA:Tom reporter expression is restricted to plasma cells. **(A)** Schematic depiction of the wildtype *Tnfrsf17* (BCMA) locus (upper) and the targeted BCMA:Tom reporter locus. Blue boxes indicate the three exons of the *Tnfrsf17* locus, and light blue boxes indicate the 5’ and 3’ untranslated region (UTR). **(B)** Flow cytometric analysis of BCMA surface abundance in splenic plasma cells. Splenocytes of BCMA:Tom reporter mice and WT controls were incubated for 18 hours in R10 medium without (ctrl) or with 1 µM DAPT and surface stained with anti-BCMA (clone 25C2) antibodies. Bars display median fluorescence intensities of anti-BCMA after secondary staining with anti-human IgG in the CD138^+^TACI^+^ antibody-secreting cell gate. (n=4-6 mice per group, Statistical analysis was performed with 2-way ANOVA with adjusted p-values (Sidak correction) for multiple comparisons), ns, not significant. **(C)** Representative flow cytometric analysis of tdTomato expression in primary and peripheral lymphoid tissues of naive BCMA:Tom mice and wildtype littermate controls. Live single cells were used as the input gate for the histograms. TdTomato-positive cells are highlighted in red in the lower panel TACI/CD138 plot. **(D)** Representative analysis of germinal center B cells (GC) and antibody-secreting cells (ASC) in mesenteric lymph nodes (mLN) of BCMA:Tom (red line histogram) and wildtype (grey filled histogram) littermates. **(E)** Light sheet microscopy of fixed and cleared intestine from WT control C57BL/6 and BCMA:Tom mice. 488 nm excitation autofluorescence is depicted in green, showing epithelial and muscle structures, and the tdTomato signal of colon and small intestine lamina propria (LP) cells is depicted in red. Left panel: side view of villi, luminal side at the bottom, smooth muscle side at the top. Right panel: top view of villi, smooth muscle side at the top. **(F)** Two-photon intra-vital microscopy of non-fixated, freshly isolated organs from WT control C57BL/6 mice and BCMA:Tom mice. Autofluorescence is depicted in white, tdTomato signal of splenic, bone marrow, mesenteric lymph node (mLN), and small intestine lamina propria (siLP) cells was directly detected and is depicted in red, green, and blue structures derived from second harmony generation (SHG) and show reflections of vessels and organ capsules.

The detection of BCMA on the surface of mouse plasma cells by flow cytometry is complicated by limited reagent availability and the fact that BCMA is shed from the cell surface by γ-secretase. Therefore, surface BCMA can only be detected on mouse plasma cells after treatment with a γ-secretase inhibitor (9). To reliably quantitate BCMA expression, we established the BCMA:Tom reporter mouse, a mouse model that expresses a tdTomato fluorescent reporter from the endogenous BCMA-encoding *Tnfrsf17* locus. Additionally, loxP sequences flanking the BCMA exon 3 were introduced to allow the conditional deletion of the terminal BCMA exon and the IRES-tdTomato cassette upon Cre-mediated recombination (**Figure 1A**). Heterozygous BCMA:Tom mice developed normally, and their homeostatic plasmablast and plasma cell numbers and serum Ig concentrations were comparable to wildtype littermates. (**Supplementary Figures S1A, B**). The introduction of the reporter cassette did not affect surface abundances of BCMA with an anti-mouse BCMA antibody (clone 25C2) (14) by flow cytometric analysis after treatment with the γ-secretase inhibitor DAPT (**Figure 1B**, **Supplementary Figures S1C, D**).

The lymphoid compartments in bone marrow (BM), spleen (SPL), mesenteric lymph nodes (mLN), thymus and peripheral blood of BCMA:Tom mice all contained a small fraction of tdTomato-positive cells (**Figure 1C**). In all analyzed compartments, the tdTomato-positive cells also produced the surface markers CD138 and TACI (**Figure 1C**). These markers precisely delineate antibody-secreting plasmablasts and plasma cells (26). In striking contrast to antibody-secreting cells (ASC), no tdTomato signal could be detected in activated germinal center B cells (**Figure 1D**) and in other non-B cell lineage populations previously described to express BCMA, e.g., T cells (27) and monocyte/macrophage populations (28) (**Supplementary Figure S1E**). We additionally observed co-localization of the tdTomato signal with cells exhibiting high intracellular kappa light chain abundance in immunofluorescence stainings of bone marrow cryo-sections, further supporting the specific labeling of ASCs by the BCMA:Tom reporter (**Supplementary Figure S1F**). While previously described plasma cell reporters are also detected outside of the plasma cell compartment in activated B cells [Jchain:CreERT2-GFP (29)] or T cells [Blimp1-GFP (10)], the BCMA:Tom mouse represents, to our knowledge, the first reporter exclusively expressed in the CD138^+^/TACI^+^ ASC compartment.

The tdTomato red fluorescent protein was selected due to its high brightness and photostability (30), which enables deep-tissue imaging applications. Using light-sheet microscopy in tissue-cleared small intestine from a BCMA:Tom mouse, we could detect BCMA:Tom-positive cells in the villi and crypts of the lamina propria (**Figure 1E**). These two locations have been identified as environments supporting long-lived plasma cells by APRIL secretion from intestinal epithelial cells (3). The tdTomato-reporter also permitted live-cell imaging without additional antibody staining across a broad spectrum of tissues harboring plasma cells in their microenvironments (**Figure 1F**).

### BCMA:Cre is active during early embryogenesis

The restricted expression of BCMA in ASCs, as observed in the BCMA:Tom reporter mouse, makes the *Tnfrsf17* locus an ideal candidate to drive the expression of a plasma cell-specific Cre recombinase. We, therefore, constructed BCMA:Cre mice with a similar CRISPR-targeting approach (**Supplementary Figure S2A**). To determine the tissue-specific activity of the BCMA:Cre deleter, we crossed it with the Ai9(RCL-tdT) mouse line containing a R26-STOP-Tomato cassette (31). Unexpectedly, we observed ubiquitous tdTomato fluorescence across all tissues, indicating a Cre-mediated excision of the loxP-flanked STOP cassette early during mouse embryonic development (**Supplementary Figures S2B, C**). A transcriptome dataset of mouse pre-implantation embryos (32) revealed *Tnfrsf17* transcripts in the 4-cell stage and early 8-cell embryos, while in advanced embryos, *Tnfrsf17* expression gradually decreased and altogether ceased in blastocysts (**Supplementary Figure S2D**). While counteracting roles of BAFF and APRIL in pregnancy have been described (33), the functional significance of BCMA expression in early embryogenesis remains unknown. Therefore, like the Blimp1-Cre mouse (34), the BCMA:Cre deleter is not suitable for restricted conditional gene ablation in mature plasma cells.

### BCMA defines mature IgG- and IgM-producing plasma cells

**Figure 2:**
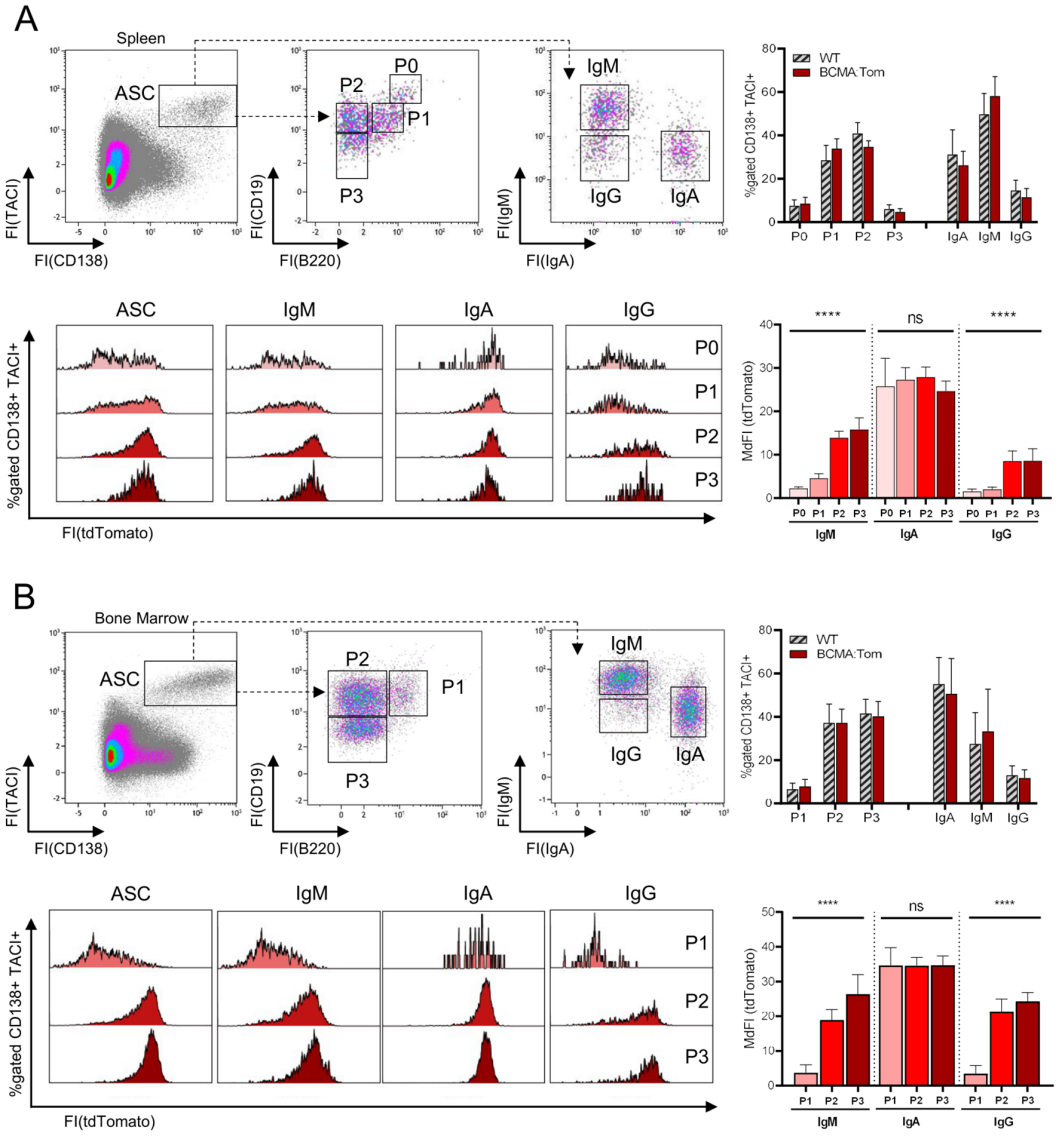
BCMA expression is linked to IgH isotype and mature plasma cell subsets. Representative flow cytometric analysis of antibody-secreting cell (ASC) subsets in **(A)** spleen and **(B)** bone marrow of BCMA:Tom reporter mice and WT littermates. TACI^+^/CD138^+^ ASCs were gated from viable lymphocytes and subdivided based on CD19/B220 and surface IgM/IgA abundance. The upper right panel depicts the frequency of the respective subpopulations within the ASC gate. tdTomato expression within the total TACI^+^/CD138^+^ gate and the IgH isotype gated subpopulations in BCMA:Tom mice is displayed in the lower panel histograms and summarized in the lower panel bar chart (mean +/- SD). Statistical analysis was performed using repeated-measures one-way ANOVA with testing for a linear trend per isotype (n=7-9 per group). ns, not significant, ****,p < 0.0001.

The tdTomato fluorescence within CD138^+^/TACI^+^ ASCs in the spleen and bone marrow showed a bi-modal distribution (**Figure 2**), indicating the existence of BCMA-negative and BCMA-positive populations. We, therefore, characterized the expression of BCMA across the previously described subpopulations defined by surface abundances of B220, CD19 and IgH isotypes (26). The proportion of BCMA-negative ASCs decreased in the spleen from the proliferating P0 (B220^hi^CD19^hi^) and P1 (B220^+^CD19^+^) plasmablasts to B220-negative mature plasma cell populations P2 (B220^−^CD19^+^) and P3 (B220^−^CD19^−^), both of which show a higher and more homogenous tdTomato fluorescence than the B220-positive P0 and P1 fractions (**Figure 2A**). Similarly, in the bone marrow, where the P0 population is absent, tdTomato fluorescence in the total ASC compartment significantly increased from P1 plasmablast to the mature P2/P3 plasma cell populations (**Figure 2B**).

However, when we additionally stratified the BCMA:Tom fluorescence in these subpopulations based on their IgH isotype, we found a differential expression of the BCMA:Tom reporter (**Figures 2A, B**). IgA plasmablasts and plasma cells (P1-P3) in the bone marrow and spleen show a higher BCMA:Tom fluorescence than IgM and IgG ASCs, with no significant differences in fluorescence intensities between the P0/P1 plasmablast stages and the mature P2/P3 stages. The observed correlation of BCMA with the plasma cell IgH isotype is consistent with previous transcriptome analyses, where IgA plasma cells reproducibly displayed the highest *Tnfrsf17* transcript abundances (**Supplementary Figure S3A**) (23, 35). These results further indicate that IgA plasma cells, typically generated in mucosal tissues, might follow a distinct differentiation trajectory influenced by environmental factors, leading to early and robust BCMA expression.

### Terminal plasma cell maturation relies on *in vivo* niche factors

**Figure 3:**
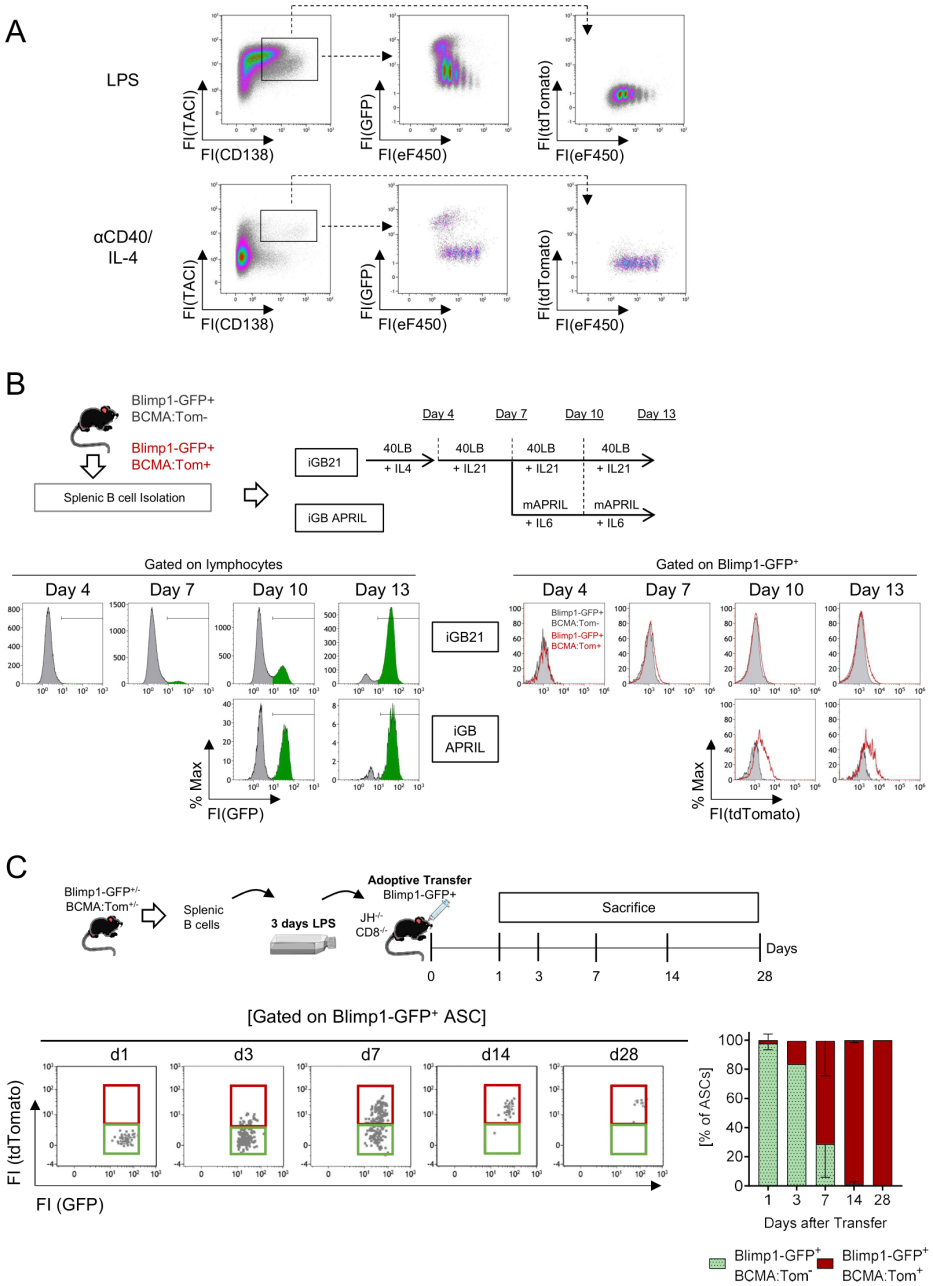
*In vitro*-generated plasmablasts only develop into mature plasma cells after transfer *in vivo.* **(A)** Splenic B cells of Blimp1-GFP/BCMA:Tom mice were magnetically isolated, labeled with the proliferation dye eFluor450 and stimulated with LPS or anti-CD40+IL-4 for 3 days. Representative data of N=3 independent experiments. **(B)** Splenic B cells of Blimp1-GFP/BCMA:Tom mice were magnetically isolated, co-cultured on 40LB-Feeder cells and stimulated with IL-4 (day 0-4), IL21 (Day 4-10) or APRIL/IL6 without Feeder cells (Day 10-13). Plasmablast differentiation was monitored by the induction of Blimp1-GFP and BCMA:Tom reporter abundance. Representative data of N=2 independent experiments with a total of 3 Blimp1-GFP^(+/−)^/BCMA:Tom^(+/−)^ and 2 Blimp1-GFP^(+/−)^/BCMA:Tom^(−/−)^ reporter mice. **(C)** Splenic B cells were magnetically isolated from Blimp1-GFP/BCMA:Tom mice and activated with LPS for 3 days. On day 3, 5 million Blimp1-GFP^+^BCMA:Tom^−^ cells were isolated by FACS and transferred into JH^−/−^ CD8^−/−^ mice. Recipient mice were sacrificed on days 1, 3, 7, 14 and 28 after adoptive transfer. Blimp1-GFP^+^CD138^+^TACI^+^ antibody-secreting cells (ASC) in the bone marrow were quantified by flow cytometry at the indicated time points after transfer. The frequency of Blimp1-GFP^+^BCMA:Tom^−^ (green) and Blimp1-GFP^+^BCMA:Tom^+^ (red) cells at each timepoint is depicted (mean ± SD). Data are shown for 3 mice per timepoint (except day 3 with only one mouse analyzed).

BCMA:Tom fluorescence was induced in the P0/P1 plasmablast population (**Figure 2**) but not in activated germinal center B cells *in vivo* (**Figure 1D**). As the transition of a B cell to a plasma cell depends on the upregulation of the transcription factor Blimp1 (36), Blimp1-GFP mice (10) were crossed with BCMA:Tom mice to generate double reporter mice that enable the tracking of both the onset of plasma cell differentiation and the dynamics of BCMA:Tom expression. To determine signals and conditions required for the induction of BCMA:Tom expression during plasma cell differentiation, isolated splenic B cells from Blimp1-GFP/BCMA:Tom mice were activated *in vitro* under T-dependent (anti-CD40/IL-4) and T-independent (LPS) conditions (**Figure 3A**).

Both stimulations strongly induced proliferation and upregulation of TACI on day 3, with detectable Blimp1-GFP fluorescence in proliferated cells, most of which showed a high abundance of CD138 (CD138^hi^). Blimp1-GFP-positive cells were readily detectable in the LPS and anti-CD40/IL-4 stimulated cultures, demonstrating robust plasmablast differentiation. However, BCMA:Tom fluorescence could not be observed under either condition (**Figure 3A**).

To test whether an *in vitro* germinal center-like B cell culture system yields mature BCMA-positive plasma cells, we co-cultured primary B cells from Blimp1-GFP/BCMA:Tom mice on BAFF/CD40L-transduced feeder cells (12) in the presence of cytokines inducing plasma cell differentiation and maintenance (**Figure 3B**). After an initial activation phase in the presence of IL-4, switching to IL-21 did not result in observable shifts in BCMA:Tom fluorescence, despite robust induction of Blimp1-GFP. Prolonged culture up to 13 days in the presence of the stimulatory feeder cells and IL-21 failed to produce a marked induction of BCMA:Tom expression.

To mimic the migration of plasmablasts into a niche micromilieu, the activated B cells were removed from feeder cells after 7 days and maintained in the presence of the pro-survival factors IL-6 and multimeric APRIL (mAPRIL) (37). Under these conditions, a subtle increase in BCMA:Tom fluorescence could be detected (**Figure 3B**). However, BCMA:Tom expression was only marginal compared to the observed signals in plasma cells *in vivo* (**Figure 2**). These findings indicate that the generation of Blimp1-GFP^+^ plasmablasts can be replicated by *in vitro* culture systems; however, terminal plasma cell differentiation marked by BCMA:Tom depends on additional factors.

Initiation of *Tnfrsf17* transcription depends on Blimp1 (38) and IRF4 (39) in murine plasmacytoma cell lines. Because Blimp1-GFP is strongly expressed in our *in vitro* cultures and IRF4 upregulation is known to precede Blimp1 induction upon plasmablast differentiation (39), the absence of BCMA:Tom fluorescence *in vitro* indicates the insufficiency of the mere presence of both transcription factors and emphasizes the need of an *in vivo* microenvironment to induce BCMA expression in plasmablasts generated *in vitro.*

To test this hypothesis, we activated B cells from Blimp1-GFP/BCMA:Tom reporter mice with LPS and adoptively transferred Blimp1-GFP^+^ plasmablasts into B cell-deficient JH^−/−^/CD8^−/−^ recipients and monitored the fate of the transferred plasmablasts in the bone marrow and spleen by flow cytometry (**Figure 3C**, **Supplementary Figures S3B, C**). One day after the transfer, the transplanted cells remained Blimp1-GFP^+^ without detectable BCMA:Tom fluorescence. The frequency of BCMA:Tom^+^ cells gradually increased between day 3 and day 14 after the transfer, resulting in more than 90% of Blimp1-GFP^+^/BCMA:Tom^+^ plasma cells in the bone marrow after 2 weeks. The BCMA:Tom fluorescence intensity observed 7 days after the transfer was comparable to the fluorescence intensity in bone marrow plasma cells from BCMA:Tom mice (**Figure 2**). These results demonstrate that mimicking T cell-independent activation of B cells *in vitro* allows the formation of mature BCMA-positive plasma cells upon transfer into B cell-deficient recipient mice. However, absolute numbers of BCMA:Tom^+^ ASCs in the bone marrow and spleen were very low by day 28 after transfer (**Supplementary Figure S3B**) and undetectable in both organs after 98 days (data not shown). Therefore, high BCMA expression alone is insufficient to establish plasma cell longevity in the marrow. Thus, the BCMA:Tom reporter is a reliable marker for more mature ASCs, but not necessarily for long-lived plasma cells.

### A distinct transcriptome defines plasma cell maturation

**Figure 4:**
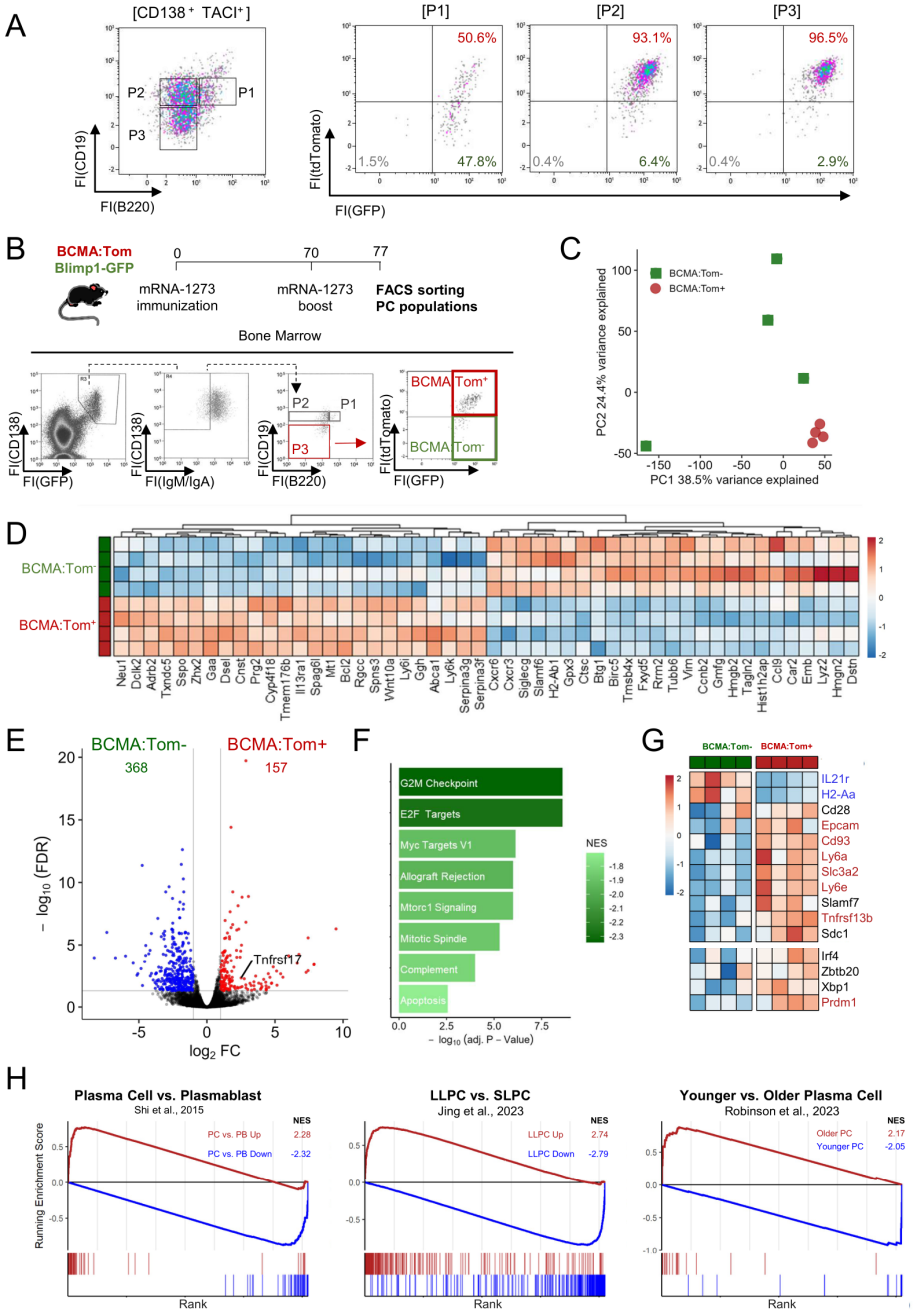
BCMA expression distinguishes a mature plasma cell transcriptome. **(A)** Bone marrow plasma cells of unimmunized Blimp1-GFP/BCMA:Tom mice were analyzed for reporter gene expression by flow cytometry. Antibody-secreting cells (CD138^+^TACI^+^) were used as input for the left-most plot and further subdivided into subpopulations based on CD19/B220 expression. **(B)** Blimp1-GFP/BCMA:Tom mice were immunized with mRNA-1273 on days 0 and 70. Surface IgM^−^/IgA^−^ P3 (B220^−^/CD19^−^) plasma cell populations were sorted for transcriptome analysis based on BCMA:Tom expression. **(C)** Principal component analysis (PCA) of transcriptome data showing the variance between samples, with the first two principal components (PC1 and PC2) accounting for the highest variance in gene expression profiles across the two sample groups. **(D)** Heatmap displaying the top 50 up-regulated and downregulated differentially expressed genes (DEGs) across samples with gene-wise color scaling indicating higher expression in red and lower expression in blue. **(E)** Volcano plot with DEGs (|log2FC| > 1 and FDR < 0.05) highlighted in red (higher in BCMA:Tom^+^) and blue (higher in BCMA:Tom^−^). **(F)** Gene Set Enrichment Analysis (GSEA) of significantly enriched MSigDB-Hallmark pathways with color-scaling by the normalized enrichment score (NES). A negative NES indicates enrichment of the respective gene set in BCMA:Tom^−^ cells. **(G)** Heatmap of selected plasma cell maturation signature genes with significantly expressed gene symbols highlighted in red/blue. **(H)** GSEA Analysis of curated plasma cell signature gene sets derived from literature [Shi et al., 2015a (40); Jing et al., 2024a (41); Robinson et al., 2023 (2)]. Enrichment scores for BCMA:Tom^+^ cells are indicated in red and for BCMA:Tom^−^ in blue.

The transfer of *in vitro* differentiated plasmablasts revealed a step-wise maturation process with Blimp1-GFP upregulation preceding BCMA:Tom induction. To test whether this sequential expression pattern is also reflected in *in vivo* ASC development, Blimp1-GFP and BCMA:Tom reporter fluorescences were analyzed within bone marrow plasma cell subpopulations (**Figure 4A**). Interestingly, Blimp1-GFP^+^/BCMA:Tom^−^ (BCMA:Tom^−^) ASCs were detectable in all plasma cell subpopulations, albeit with a decreasing frequency from the B220^+^/CD19^+^ P1 population to the B220^−^/CD19^−^ P3 population. This aligns with the established concept of increased maturation, which is marked by the loss of B cell markers B220 and CD19. However, the expression patterns of the Blimp1-GFP and BCMA:Tom reporters indicate additional heterogeneity within these ASC populations.

As BCMA:Tom^−^ plasmablasts converted into BCMA:Tom^+^ plasma cells within days after transfer, the BCMA:Tom^−^ cells likely represent ontogenetically younger precursors that have recently entered the bone marrow for terminal maturation into plasma cells. To examine the characteristics of these cells, we performed transcriptome analysis by RNA sequencing. The BCMA:Tom^−^ cells are scarce in homeostasis. We therefore immunized Blimp1-GFP/BCMA:Tom mice with the SARS-CoV-2 mRNA vaccine mRNA-1273 and boosted 70 days later to induce infiltration of plasmablasts into the bone marrow (**Figure 4B**). Seven days after the boost, BCMA:Tom^−^ and BCMA:Tom^+^ P3 plasma cells were sorted from the bone marrow. We analyzed the pre-defined P3 plasma cell population because these cells have been characterized as the most mature subpopulation of ASCs (26). To account for the diverging transcriptomes linked to the IgH isotype (23), only IgG plasma cells were isolated by gating on surface IgA^−^/IgM^−^ P3 plasma cells (**Figure 4B**). Repertoire reconstruction confirmed the purity of the sorted plasma cells with >90% of clones detected with IgG constant regions (**Supplementary Figure S3C**).

Principal component analysis revealed a distinct clustering of BCMA:Tom^+^ plasma cells, indicating a conserved transcription profile among the fully matured plasma cells. In contrast, the transcriptomes of BCMA:Tom^−^ cells displayed a higher variability, consistent with the assumption that they contain a continuum of precursor stages transitioning towards a mature phenotype (**Figure 4C**). By comparing the BCMA:Tom^+^ to the BCMA:Tom^−^ population, 157 genes were up-regulated and 368 genes were down-regulated in BCMA:Tom^+^ cells, including elevated *Tnfrsf17* abundances (encodes BCMA) in the tdTomato-expressing plasma cells (**Figure 4E**, **Supplementary Table 1**). The top up-regulated genes in BCMA:Tom^−^ cells encoded proliferation-associated proteins (e.g., *Birc5, Hmgb2, Ccnb2*), which is consistent with the robust proliferative capacity highlighted by enrichment of hallmark pathways regulating cell cycle (**Figures 4D, F**). The increased expression of migration factors (*Cxcr3, Cxcr6, Fxyd5, Gmf*g) in the BCMA:Tom^−^ population further characterizes these cells as newly arriving plasmablasts in the bone marrow.

The BCMA:Tom^−^ cells also exhibited elevated expression for the surface markers *Slamf6* and MHC-II (*H2-Ab1*), which have recently been associated with an immature plasma cell phenotype (2). Other established markers of plasma cell maturation followed the expected patterns (**Figure 4G**), with a decrease of the germinal center-associated *IL-21R* and upregulation of plasma cell-associated *Epcam, Cd93, Slc3a2* and *Ly6a/e* in the BCMA:Tom^+^ plasma cells. Both BCMA:Tom^−^ and BCMA:Tom^+^ cells clearly expressed key plasma cell transcription factors, suggesting that both populations maintain the regulatory machinery required for plasma cell function. However, significantly increased expression of the plasma cell master transcription factor Blimp1 (*Prdm1*) could be detected in BCMA:Tom^+^ plasma cells, underscoring the advanced maturation of these cells (10, 26).

Gene signatures of plasmablasts and plasma cells (40) and long-lived plasma cells (LLPC) identified by genetic time-stamping have been described (2, 41). Comparing these gene sets with the observed transcriptional changes upon BCMA:Tom-induction, we could demonstrate a strong enrichment of up-regulated genes in the BCMA:Tom^+^ group with gene signatures derived from mature plasma cells. Conversely, transcriptomes from BCMA:Tom^−^ cells correlated with signatures of immature plasmablasts (**Figure 4H**). Therefore, we conclude that BCMA induction marks a distinct step in the terminal maturation of plasma cells in the bone marrow. Despite the convincing enrichment of a long-lived plasma cell gene signature in the BCMA:Tom^+^ compartment, we cannot assume that all BCMA:Tom^+^ cells are long-lived. The enrichment rather implies that long-lived plasma cells share the distinct transcriptome of mature plasma cells.

**Figure 5:**
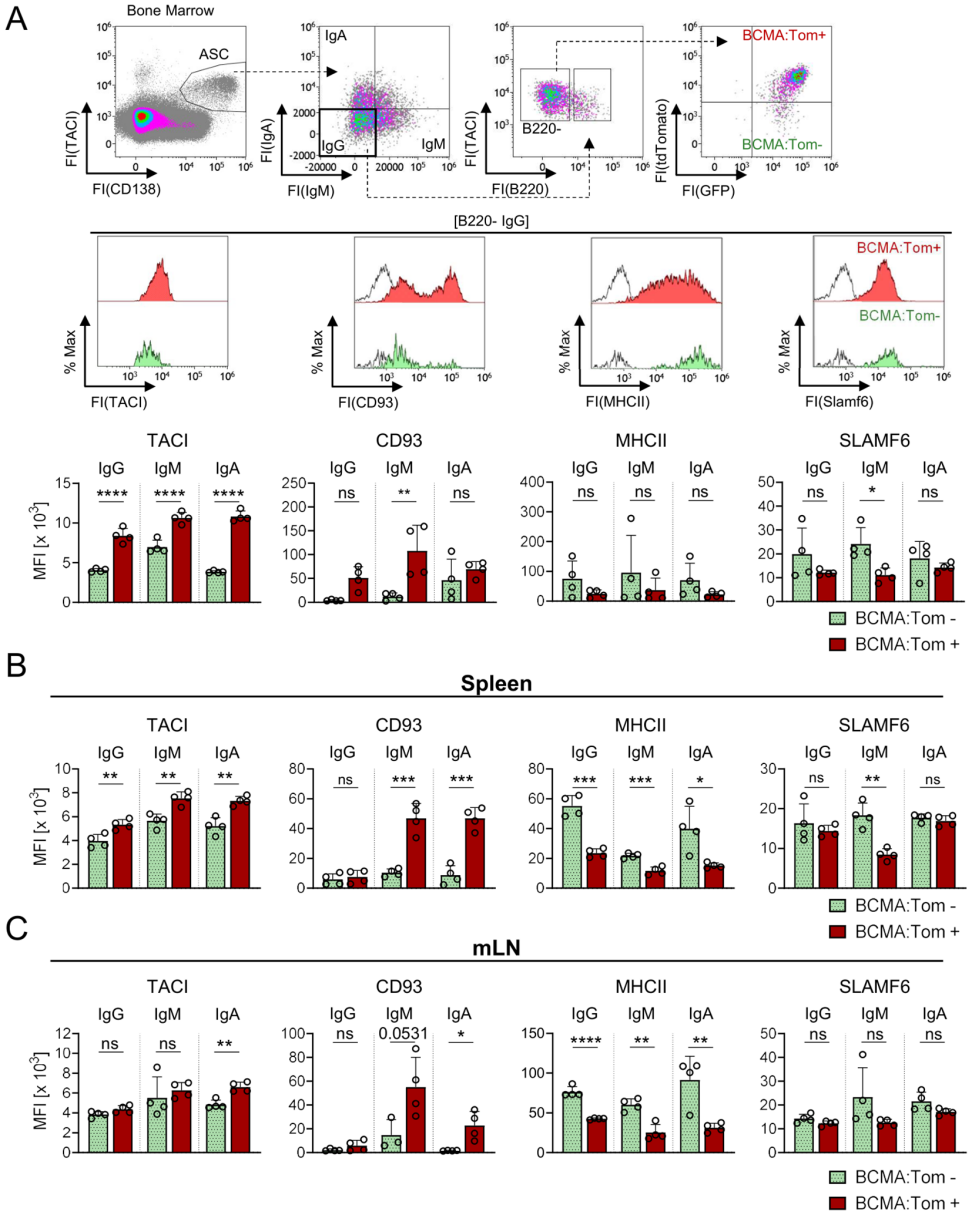
BCMA expression correlates with surface marker abundance across B220^−^ ASC isotypes and tissues. Selected markers were validated at the protein level by flow cytometry on **(A)** B220^−^/IgM^−^/IgA^−^ BCMA:Tom^−^ (green) and BCMA:Tom^+^ (red) bone marrow plasma cells of unimmunized Blimp1-GFP/BCMA:Tom mice. Open histograms represent the FMO controls. The surface abundance of selected markers was further analyzed for both populations on B220^−^/IgM^+^ or B220^−^/IgA^+^ ASCs in the bone marrow. **(B, C)** The protein abundance of selected markers of BCMA:Tom^−^ and BCMA:Tom^+^ ASC for the Ig heavy chain isotypes IgG, IgM and IgA are summarized in **(B)** spleen and **(C)** mLN. Median fluorescence intensities (MFI) are summarized in bar charts (mean +/- SD). Statistical analysis was performed using two-way ANOVA with Šídák’s multiple comparisons test (n=4 per group). ns, not significant, p > 0.05; *, p < 0.05; **, p <0.01; ***, p < 0.001; ****, p < 0.0001.

To validate our findings from the transcriptomic analysis of BCMA:Tom^−^ and BCMA:Tom^+^ ASCs in the bone marrow, we analyzed the surface abundance of selected maturation markers across the ASC compartments in the bone marrow, spleen, and mesenteric lymph nodes (mLN) for mature B220^−^ ASC (**Figure 5**) and immature B220^+^ ASC (**Supplementary Figure S4**). We focused on CD93, which has been described as a factor of plasma cell persistence with elevated abundance in mature ASCs (42). Additionally, we analyzed SLAMF6 (Ly108) and MHC class II (I-A/I-E) within B220^−^ and B220^+^ ASC populations stratified by IgH isotype expression, as these markers have been recently identified to be downregulated in aged ASCs (2).

CD93 displayed a bi-modal expression pattern in mature B220^−^ IgG ASCs of the bone marrow, with BCMA:Tom^+^ cells showing higher mean fluorescence intensity (MFI) due to an increased proportion of CD93^+^ cells (**Figure 5A**). In contrast, TACI B220^−^/BCMA:Tom^+^ population, while MHCII and SLAMF6 abundances were reduced in BCMA:Tom^+^ cells, without reaching statistical significance. Similar trends were observed in B220^−^/IgM^+^ and B220^−^/IgA^+^ ASCs within the bone marrow, indicating conserved maturation dynamics across isotypes (**Figure 5B**). In B220^+^ ASCs, TACI and CD93 displayed similar expression trends as observed in B220^−^ ASCs, with BCMA:Tom^+^ cells showing higher CD93 abundances and elevated TACI expression (**Supplementary Figure S4A**). However, the differences between BCMA:Tom^−^ and BCMA:Tom^+^ cells were less pronounced in the B220^+^ than in the B220^−^ ASC population.

In the spleen and mLN, the surface marker expression profiles demonstrated both similarities and differences compared to that found in the bone marrow (**Figures 5B, C**). BCMA:Tom^+^ IgG ASCs in the spleen and mLN did not show an increase in CD93 abundance, in contrast to their bone marrow counterparts. However, BCMA:Tom^+^ ASCs producing IgA and IgM exhibited a pronounced shift toward higher CD93 levels in the spleen and mLN. For MHCII and SLAMF6, the expression profiles in mature B220^−^ ASCs were consistent with the bone marrow, showing a reduced surface abundance in BCMA:Tom^+^ cells, with a substantial loss of surface MHCII observed in these tissues upon induction of BCMA. Interestingly, the reduction of SLAMF6 surface abundances remained strongest in the IgM^+^ ASC subsets, in line with the observed pattern in the bone marrow. The immature B220^+^ ASC compartments in the spleen and mLN displayed similar surface abundances of the analyzed markers between BCMA:Tom^−^ and BCMA:Tom^+^ cells, except for increased CD93 abundances on splenic IgA/IgM ASCs correlating with the induction of BCMA (**Supplementary Figures S4B, C**).

The findings from the flow cytometry analyses confirmed the differences identified in the transcriptome analysis of bone marrow IgG-producing ASCs and extended the observations to IgM^+^ and IgA^+^ subsets. Furthermore, they revealed tissue-specific differences in the regulation of surface markers, particularly for CD93 and SLAMF6, underscoring the interplay between tissue microenvironments and IgH isotype in shaping plasma cell maturation.

## Discussion

This study introduces the BCMA:Tom reporter mouse as a robust tool for tracking plasma cell maturation and BCMA expression dynamics. We demonstrated that mouse BCMA is exclusively expressed by ASCs and that its expression level correlates with a mature plasma cell transcriptome in IgG-producing plasma cells. The BCMA:Tom mouse represents, to our knowledge, the first reporter that is exclusively expressed in the ASC compartment, overcoming limitations of previously described plasma cell reporters that are expressed outside of the plasma cell compartment in activated B cells (Jchain:CreERT2-GFP (29) or T cells [Blimp1-GFP (10)]).

Although BCMA expression is restricted to ASCs in adult mice, the BCMA:Cre deleter mouse unexpectedly revealed transient *Tnfrsf17* induction during pre-implantation embryogenesis. *Tnfrsf17* transcripts were detected at the 4-cell and early 8-cell stages, indicating a developmental role unrelated to plasma cell biology. This early activity limits the specificity of BCMA:Cre for mature plasma cells but highlights an unexpected aspect of *Tnfrsf17* regulation during embryogenesis, meriting further exploration of the function of BCMA and its ligand APRIL in early development. Within the ASC compartment, BCMA shows a bimodal expression pattern and is differentially expressed by plasma cells with different IgH isotypes, with the highest tdTomato fluorescence observed in IgA plasma cells. The observed correlation of BCMA with the plasma cell IgH isotype aligns with prior transcriptome analyses, where IgA plasma cells reproducibly displayed the highest *Tnfrsf17* transcript abundances (**Supplementary Figure S3A**) (23, 35). This association likely reflects the micromilieus of their induction sites, with the majority of IgG and IgM plasma cells generated in the spleen and lymph nodes and IgA plasma cells primarily originating from mucosal tissues (4). We observed elevated BCMA expression already among B220^+^/IgA^+^ ASC in the bone marrow. This may indicate intrinsic differences in BCMA regulation and/or plasma cell maturation in general between IgA^+^ and other IgH-isotype-expressing ASCs. The induction of BCMA early in the IgA ASC ontogeny might be driven by distinct signaling cues encountered in their mucosal induction sites, such as TGF-β, retinoic acid, or microbiota-derived signals. The differential expression pattern of BCMA in ASC of different IgH-isotypes underscores the importance of local environmental factors in shaping the maturation and functional properties of plasma cells.

The incomplete induction of BCMA in various *in vitro* stimulation conditions further emphasized the importance of the tissue environment for plasma cell maturation. While the *in vitro* culture systems effectively generated Blimp1-GFP^+^ plasmablasts, terminal plasma cell differentiation marked by BCMA:Tom remained dependent on additional factors absent in these settings. These missing factors may include contacts with stromal cells, availability and/or timing of cytokine exposures, metabolites, matrix components, hypoxia or a combination of factors required to mimic a micromilieu enabling the maturation of plasma cells (43). Although the survival-promoting cytokines APRIL and IL-6 support plasma cell survival *in vitro* (44), both are insufficient to mediate maturation comparable to the resting P2/P3 plasma cell subpopulations *in vivo*. The inability to complete the induction of BCMA:Tom highlights the shortcomings of these *in vitro* systems in recapitulating the environment required for terminal plasma cell differentiation.

The specific factors provided by the *in vivo* microenvironment induced BCMA:Tom within 3 to 7 days after transfer with a previous 3-day *in vitro* pre-stimulation period, indicating a time-window of 10-13 days required to complete the maturation from proliferating plasmablasts into resting, mature plasma cells. This aligns with previous reports showing that the differentiation of mature murine plasma cells *in vitro* takes more than 8 days (45). Although *in vitro*v-activated LPS plasmablasts developed into phenotypically BCMA:Tom^+^ mature plasma cells after transfer into B cell-deficient recipients, no long-lived plasma cell compartment was established in the bone marrow, with only scant numbers of BCMA:Tom^+^ plasma cells recoverable 28 days after transfer. This points towards intrinsic mechanisms that control the longevity of these cells, as the B cell-deficient recipients should offer undisputed bone marrow niches capable of sustaining long-lived plasma cells (46). Therefore, BCMA alone is not sufficient to establish plasma cell longevity in the marrow; thus, the BCMA:Tom reporter is a marker of a mature plasma cell population but not necessarily for long-lived plasma cells.

In summary, we established the BCMA:Tom mouse model as the first reporter exclusively labeling CD138^+^/TACI^+^ ASC and enabling the monitoring of BCMA expression dynamics. Within the ASC compartment, BCMA expression varied with the IgH isotype and increased with maturation in IgG- and IgM-producing ASCs. Therefore, the BCMA:Tom reporter, together with surface markers, IgH isotypes and the Blimp1-GFP-reporter, enables the complementation of the definitions of ASC subpopulations and the resolution of plasma cell maturation within survival niche-containing tissues, advancing our understanding of the pre-requisite processes to establish a durable humoral immunity.

## Data Availability

The datasets presented in this study can be found in online repositories. The names of the repository/repositories and accession number(s) can be found below: https://www.ncbi.nlm.nih.gov/geo/, GSE276846.
