# Supplementary material for: Decoding plasma cell maturation dynamics with BCMA
Source: Front Immunol. 2025 Mar 11;16:1539773. doi: 10.3389/fimmu.2025.1539773 (PMC11932843; doi:10.3389/fimmu.2025.1539773)
Supplement: Supplementary file 1 [file DataSheet1.pdf]

SUPPLEMENTAL MATERIAL

Schulz et al.

Decoding Plasma Cell Maturation Dynamics with BCMA

Supplemental Figures

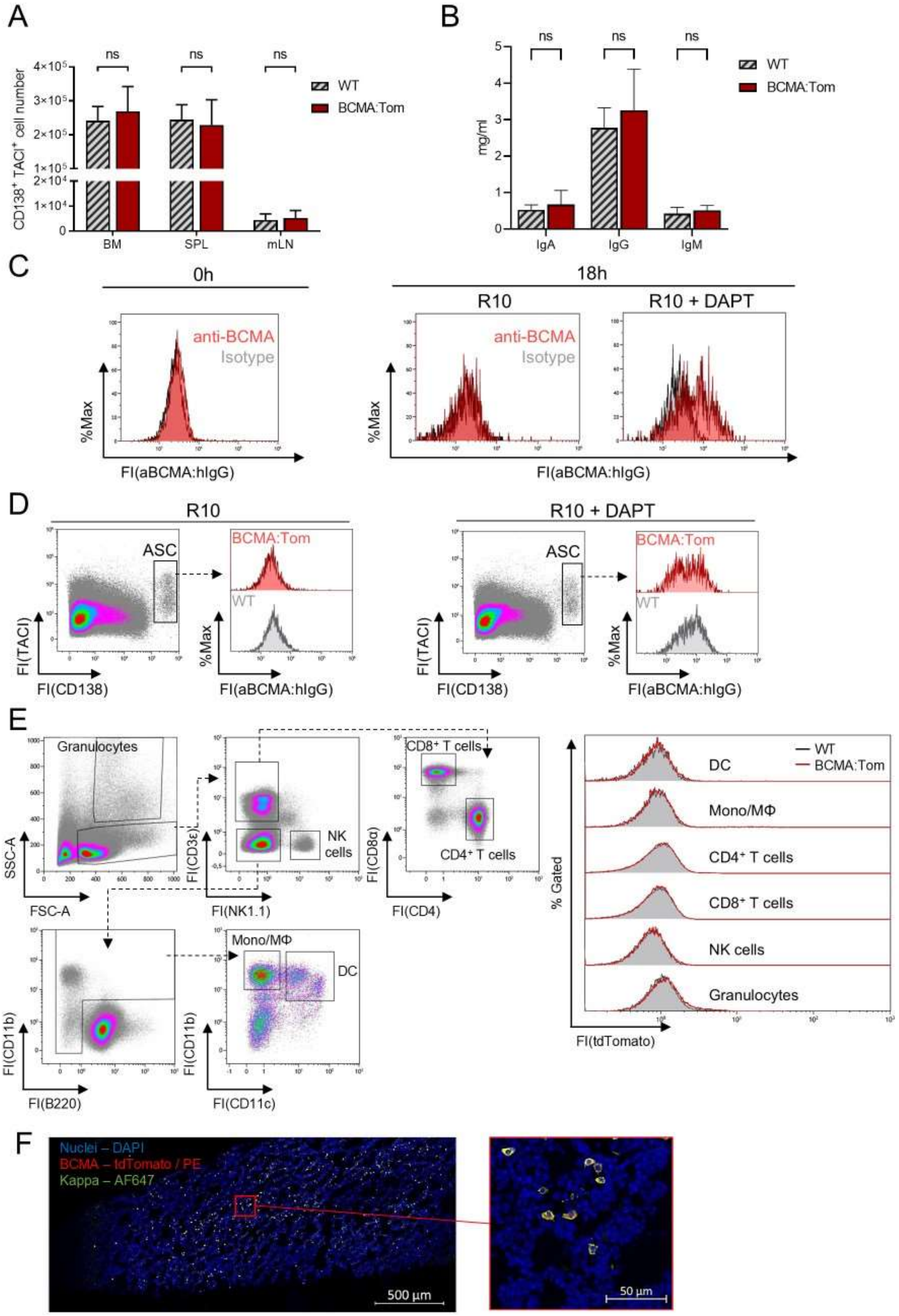

Supplementary Figure 1. Characterization of BCMA:Tom mice.

Supplementary Figure 1. Characterization of BCMA:Tom mice. (A) Absolute numbers of CD138$^+$ TACI$^+$ antibody-secreting cells (ASC) in the bone marrow (BM), spleen (SPL) and mesenteric lymph node (mLN) of unimmunized BCMA:Tom mice and wildtype littermate controls (n=7-9 mice per group). (**B)** Serum concentrations of IgA, IgG and IgM of unimmunized BCMA:Tom mice and wildtype littermate controls (n=7-9 mice per group). Statistical analysis was performed using repeated-measures 2-way ANOVA with adjusted p-values (Sidak correction) for multiple comparisons. ns, not significant $p > 0.05$. (**C)** Bone marrow of Blimp1-GFP reporter mice was isolated and stained with anti-BCMA (clone 25C2) or the isotype control antibody (clone TRES480) before (0h) and after culture for 18h at 37°C/5% CO2 in R10 medium with the γ-secretase inhibitor DAPT (1 µM) or R10 control medium. Overlayed histograms display fluorescence intensities secondary staining with anti-human IgG in the CD138$^+$Blimp1-GFP$^+$ ASC gate. (**D)** Representative flow cytometric analysis of BCMA surface expression in splenic ASCs. Splenocytes of BCMA:Tom reporter mice and WT controls were isolated and surface stained with anti-BCMA (clone 25C2) after 18h at 37°C/5% CO2 in R10 medium with 1 µM DAPT or R10 control medium. Histograms display fluorescence intensities of anti-BCMA after secondary staining with anti-human IgG in the ASC gate. (**E)** Representative gating strategy to characterize tdTomato reporter activity in major splenic immune cell lineages with overlayed histograms of tdTomato fluorescence intensity in BCMA:Tom (red) and wildtype (black) littermates in the gates described in the upper panel. NK = natural killer cells; Mono/MΦ = monocytes/macrophages; DC = dendritic cells. **(F)** Immunofluorescence of BCMA:Tom bone marrow cryo-sections. Fixed femoral bone marrow was stained with anti-mouse Kappa-AF647 (green) and rabbit-anti-RFP with an anti-rabbit-IgG PE-labeled secondary antibody (red). DAPI (blue) was added as a nuclear staining. Overlap between Kappa and tdTomato/PE Signal is depicted in yellow.

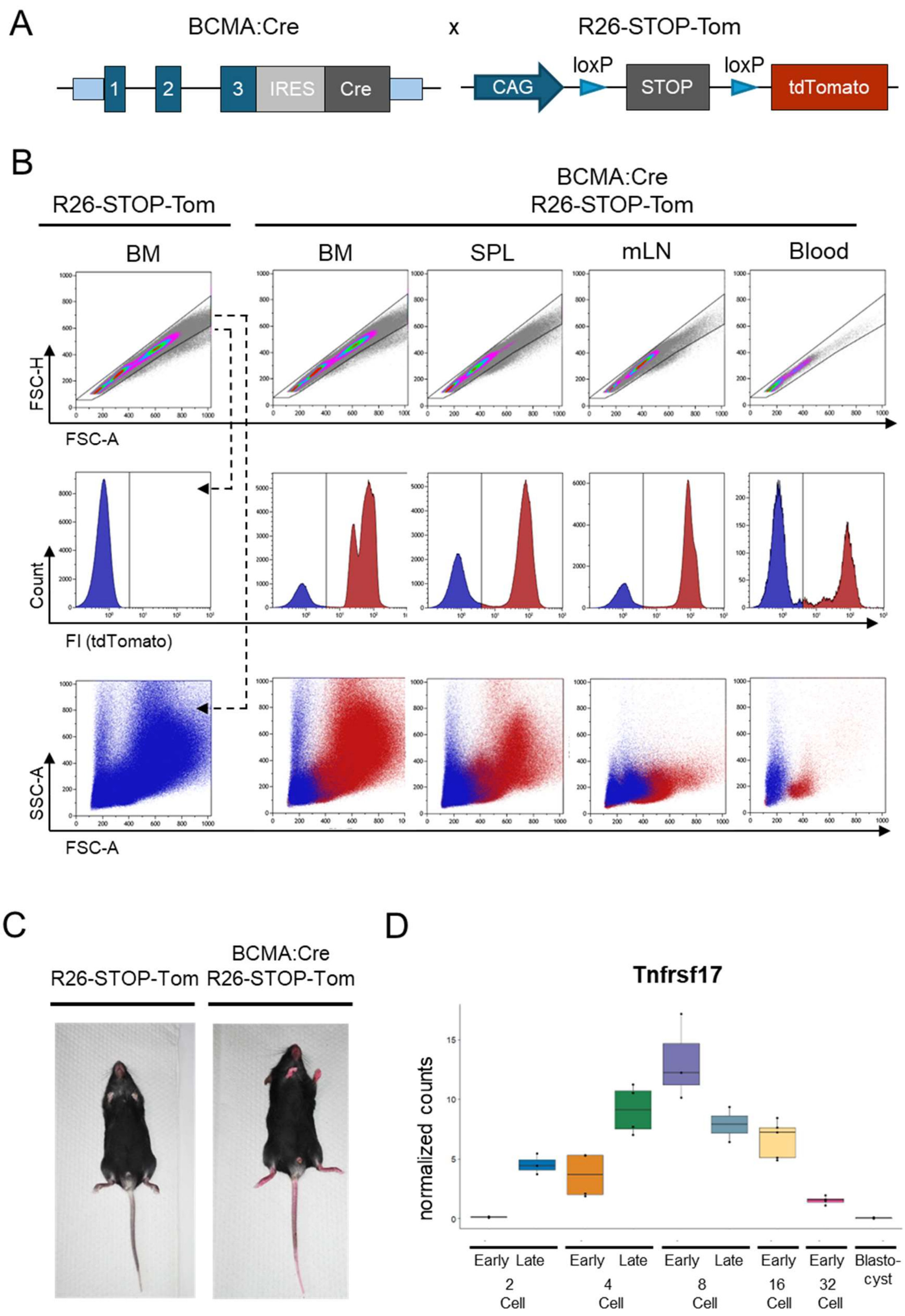

Supplementary Figure 2. BCMA:Cre deletes during early embryogenesis.

Supplementary Figure 2. BCMA:Cre deletes during early embryogenesis. (A) Schematic representation of the BCMA-Cre and R26-STOP-Tom (Ai9(RCL-tdT)) loci. (**B)** Flow cytometric analysis of bone marrow, spleen, mesenteric lymph nodes and blood of BCMA-Cre$^{(+/-)}$/R26-STOP-Tomato mice or littermate R26-STOP-Tomato controls (representative results for n=3 animals). Singlets were gated based on FSC-H/FSC-A parameters and used as input for the tdTomato histogram and FSC/SSC dot-plot with tdTomato-positive events highlighted in red and -negative events colored in blue. (**C)** BCMA-Cre$^{(+/-)}$/R26-STOP-Tomato mice show a bright red discoloration indicating deletion of the STOP-cassette in early progenitor cells. (**D)** Re-analysis of published RNA-Seq data of mouse pre-implantation embryos shows BCMA (*Tnfrsf17*) expression during early embryogenesis. The expression data was obtained from the GEO repository (GSE159484, Chi and Banerjee, 2020) and displayed as a boxplot of DESeq2-normalized counts).

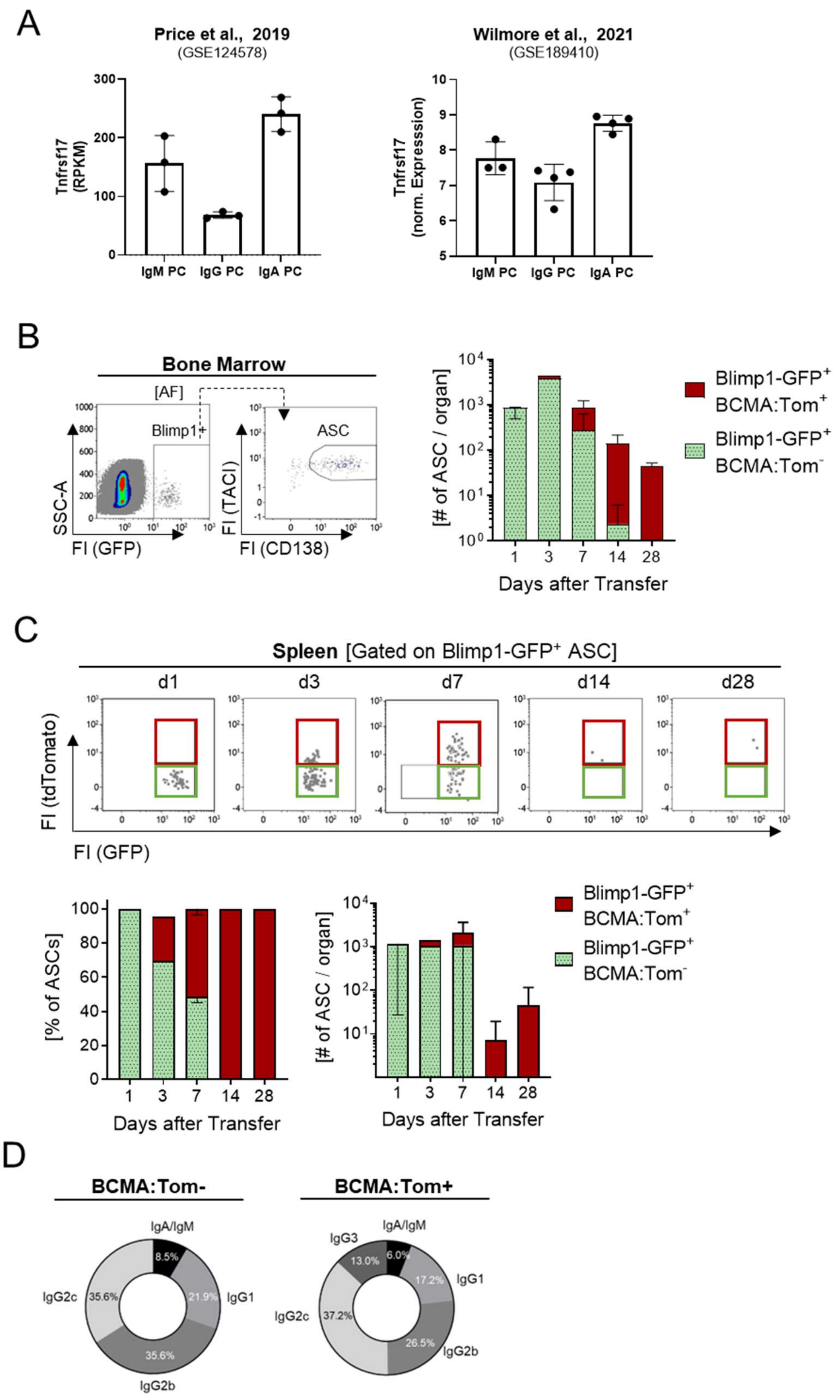

Supplementary Figure 3. BCMA expression and IgH isotype frequencies in plasma cell transcriptomes.

Supplementary Figure 3. BCMA expression and IgH isotype frequencies in plasma cell transcriptomes. (A) Expression of BCMA (*Tnfrsf17*) in published transcriptomes of IgH isotype-sorted plasma cells from spleen (Price et al., 2019) and bone marrow (Wilmore et al., 2021). (**B)** Exemplary gating strategy to identify Blimp1-GFP-positive antibody-secreting cells (ASC) in the bone marrow of recipient mice after transfer and quantification of absolute cell numbers of Blimp1-GFP$^+$BCMA:Tom$^-$ (green) and Blimp1-GFP$^+$BCMA:Tom$^+$ (red) cells in bone marrow isolated from 2 femur and 2 tibiae of the recipient mice (mean ± SD). Data shows n=3 mice/timepoint (except day 3 with one mouse analyzed). (**C)** Blimp1-GFP$^+$CD138$^+$TACI$^+$ ASCs in the spleen were quantified by flow cytometry at the indicated time points after transfer. The frequency and absolute number of Blimp1-GFP$^+$BCMA:Tom$^-$ (green) and Blimp1GFP$^+$BCMA:Tom$^+$ (red) cells at each timepoint are depicted (mean ± SD). Data shows n=3 mice/timepoint (except day 3 with one mouse analyzed) **(D)** Mean frequencies of IgH-constant regions among clones extracted by MiXCR from BCMA:Tom$^-$ and BCMA:Tom$^+$ IgG plasma cell transcriptomes (n=4 per group).

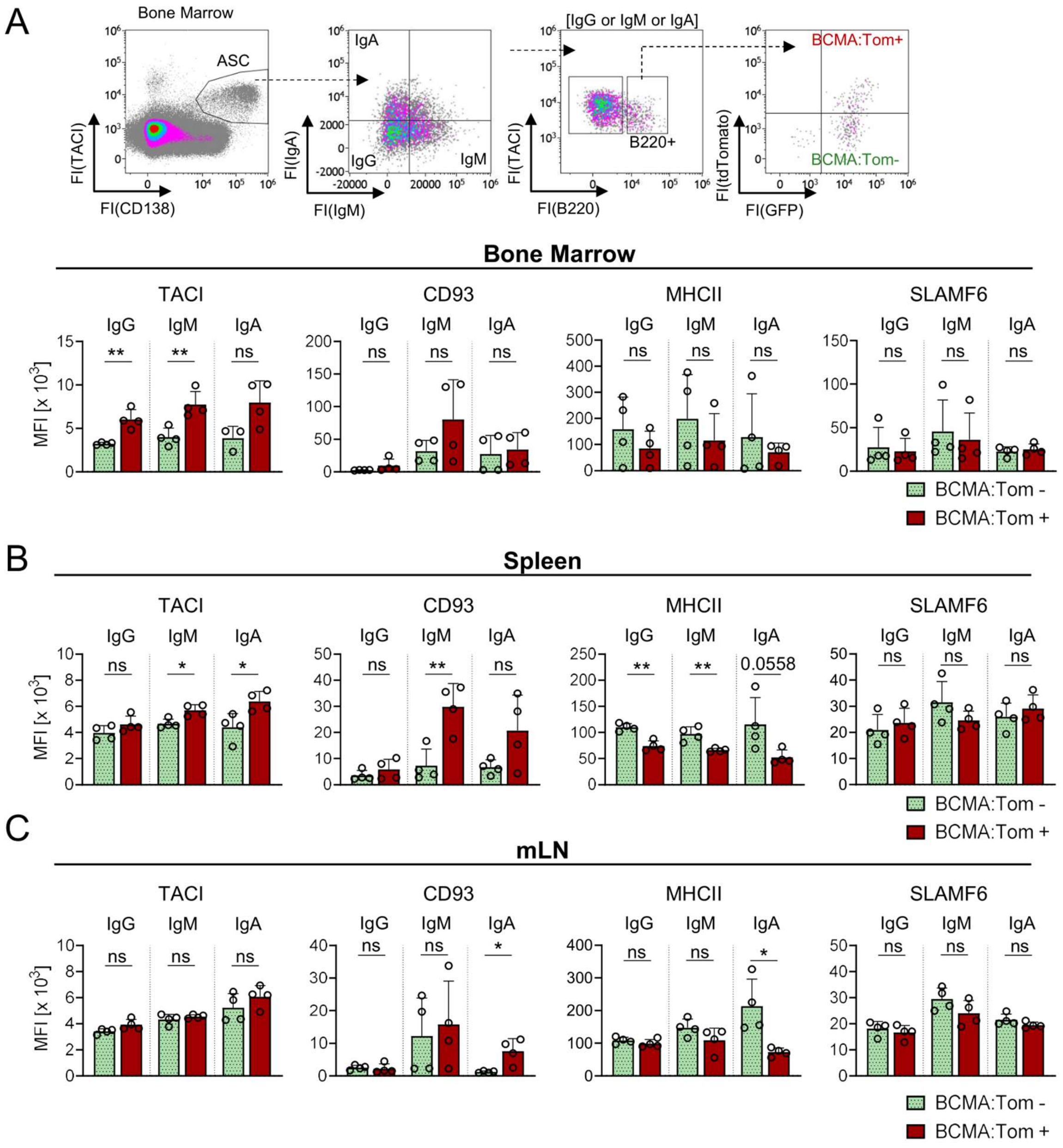

Supplementary Figure 4. BCMA expression correlates with surface marker abundance across B220^{+} ASC isotypes and tissues. (A) Selected markers were analyzed by flow cytometry on BCMA:Tom^{-} (green) and BCMA:Tom^{+} (red) B220^{+} ASC in the bone marrow of unimmunized Blimp1-GFP/BCMA:Tom mice. The surface abundance of selected markers was analyzed for IgG-, IgM- or IgA-positive ASCs in the bone marrow. **(B), (C)** The protein abundance of selected markers of BCMA:Tom^{-} and BCMA:Tom^{+} ASC for the Ig heavy chain isotypes IgG, IgM and IgA are summarized in **(B**) spleen and **(C)** mLN. Median fluorescence intensities (MFI) are summarized in bar charts (mean ± SD Statistical analysis was performed using repeated-measures 2-way ANOVA with adjusted p-values (Sidak correction) for multiple comparisons (n=4 per group). ns = not significant, p > 0.05; *, p < 0.05; **, p <0.01; ***, p < 0.001; ****, p < 0.0001.
